# Numerical Investigation of the Bending, Torsional, and Hydrostatic Pressure Responses of Hybrid Kenaf/Flax/Glass Fiber Composite Shell Structures for Unmanned Maritime Vehicles

**DOI:** 10.3390/ma19020411

**Published:** 2026-01-20

**Authors:** Yang Huang, Mohamed Thariq Hameed Sultan, Andrzej Łukaszewicz, Jerzy Józwik, Khairunnisak Latiff

**Affiliations:** 1 Department of Aerospace Engineering, Faculty of Engineering, Universiti Putra Malaysia, Serdang 43400, Selangor, Malaysia; huangyang821210@hotmail.com; 2 College of Water Resources and Coastal Engineering, Beibu Gulf University, Qinzhou 535011, China; 3 Laboratory of Biocomposite Technology, Institute of Tropical Forestry and Forest Products (INTROP), Universiti Putra Malaysia, Serdang 43400, Selangor, Malaysia; 4 Aerospace Malaysia Innovation Centre (944751-A), Prime Minister’s Department, MIGHT Partnership Hub, Jalan Impact, Cyberjaya 63000, Selangor, Malaysia; 5 Institute of Mechanical Engineering, Faculty of Mechanical Engineering, Bialystok University of Technology, 45C Wiejska St., 15-351 Bialystok, Poland; 6 Department of Production Engineering, Faculty of Mechanical Engineering, Lublin University of Technology, Nadbystrzycka St. 36, 20-618 Lublin, Poland; j.jozwik@pollub.pl; 7 School of Business and Economics, Universiti Putra Malaysia, Serdang 43400, Selangor, Malaysia; nisak@upm.edu.my

**Keywords:** fiber-reinforced composite, finite element analysis, unmanned maritime vehicle, marine application, kenaf fiber, flax fiber, glass fiber

## Abstract

Recently, with concern for the environment and the request for sustainable materials, more researchers and manufacturers have focused on the substitute solution of synthetic fiber reinforcement composites in industry applications. Green hybrid composites with natural components can present excellent sustainability, possess superior mechanical behavior, and reduce hazards. Hybridization technology allows new materials to inherit their raw materials’ characteristics and generate new properties. The current study designed novel double-walled shell structures (DS1R4L, DS2R8L, and DS5R12L), containing two thin walls and different numbers of ring and longitudinal stiffeners, as unmanned maritime vehicle (UMV) components. A normal single-walled cylindrical shell was used as a control. These models will be made of hybrid kenaf/flax/glass-fiber-reinforced composites, GKFKG and GFKFG, created in the ANSYS Workbench. The mechanical responses (deformation, stress, and strain characteristics) of models were examined under three loading conditions (end force, end torque, and hydrostatic pressure) to evaluate the influence of both material change and structural configuration. Compared to the single-walled structure, the double-walled configurations display minimized deflection and torsional angle. Moreover, GKFKG-made structures are better than GFKFG-made ones. The research contributes positively to advancing the application of hybrid kenaf/flax/glass-fiber-reinforced composites in UMV structures and promotes the development of green sustainable materials.

## 1. Introduction

Unmanned systems have experienced accelerated development due to the dual momentum of rapid advancements in communication technology and artificial intelligence [1]. Currently, unmanned equipment is extensively used on the ground, in the marine environment, and in the air, emerging into various human active fields [2,3,4]. In addition, they can help humans complete risky work. Under the support of 5G communication technology, unmanned equipment has played a prominent role in industries such as mining and aquaculture [3,4]. Nearly 70% of Earth’s surface is covered by water, yet we have not fully explored these aquatic environments [5]. Some conditions in aquatic environments are dangerous. Unmanned marine vehicles (UMVs) are automated equipment designed to replace humans performing numerous hazardous tasks in marine environments. UMVs serve as human beings’ eyes and arms to explore, manage, and protect crucial aquatic environments [5]. Moreover, they can be used in military fields such as data collection, target tracking, and mine clearance [6]. UMVs also benefit by reducing human risk, enhancing efficiency, and saving costs in maritime military missions [1].

Generally, metals are important manufacturing materials, but they are heavy, involve complex processes, are time-consuming, and require sophisticated specialized equipment. Lightweight design often plays a crucial role in enhancing product performance and reducing energy consumption. Fiber-reinforced composite materials (FRCs) are novel materials compared to the traditional ones; the materials were first produced after World War II [7]. FRCs are typically composed of two or more components: a matrix, reinforcing materials, and fillers [8]. With the synergistic effects of these components, FRCs exhibit high performance and low density, making them highly favored for weight-sensitive products. As the key component, reinforcing fiber has long been dominated by synthetic fiber (including glass fiber, carbon fiber, aramid fiber, etc.) [8]. However, these synthetic fibers can bring environmental hazards during their production and disposal procedures [9,10]. Natural fibers, with excellent mechanical properties and eco-friendly characteristics, are potential alternatives to synthetic fibers [11]. Meanwhile, they feature low cost and density, renewability, degradability, less hazardousness, and resource-rich properties, garnering more attention in the field of composite materials [12,13].

Cellulose fiber is a common type of natural fiber. These cellulose fibers can be classified as bast, fruit, grass, or seed fibers, depending on their extractive location on the plant [14]. Kenaf fiber and flax fiber are bast fibers with excellent mechanical performance, and both of them have been widely cultivated around the world and have considerable annual yields [15,16,17]. In the process of making green sustainable FRCs based on plant fibers, many scholars have made outstanding contributions. Rozyanty et al. [18] compared the water absorption and mechanical properties of biocomposites, which were fabricated from water-retted and mechanical-retted kenaf fibers, and they found that the water-retted kenaf-fiber-reinforced composites exhibited better performance in reduced water absorption and enhanced tensile properties. Singh et al. [19] found that composite materials manufactured by compression molding using woven kenaf fiber and polylactic acid (PLA) exhibited maximum tensile and flexural strengths when the fiber volume fraction was 35% under a molding temperature of 170 °C. Compared to single-plant-fiber composites, multi-fiber composite materials can bring more specific properties [20,21]. Malik et al. [22] investigated the mechanical change of pure FRCs and hybrid FRCs, which were reinforced by kenaf fiber and flax fiber under both single and hybrid configurations. They discovered that changing stacking sequences significantly influenced the mechanical properties of the hybrid composites. Hybridizing natural fibers with glass fibers can significantly enhance the mechanical properties of FRCs [23]. Huang et al. [24] assessed kenaf/flax/glass-fiber-reinforced hybrid composites vs. pure kenaf fiber composite. In the study, GFKFG displayed higher tensile strength and flexural strength than KKKKK, and GKFKG exhibited moisture absorption rates lower than KKKKK in distilled water and artificial seawater, respectively. Nowadays, the requirement for novel green materials is encouraging endless research and development of cellulose fibers in composite materials and structural applications [10,25,26].

For thin-walled structures, particularly cylindrical shells and tubes, generalized beam theory (GBT) and geometrically nonlinear shell theory are recognized as effective tools for capturing complex deformation mechanisms, including in-plane warping, cross-sectional ovalization, and nonlinear instability effects [27,28]. Silvestre [29] proposed a GBT-based method for the analysis of cylindrical hollow structures and demonstrated good agreement with finite element analysis (FEA) results. Additionally, Iandiorio & Salvini [28] employed a nonlinear shell theory that accounts for large displacements and rotations to identify in-plane cross-sectional ovalization in open and closed tubular shell structures, and the results were also validated against FEA. However, these analytical approaches still face practical limitations when dealing with the complex analysis of double-layer cylindrical shell structures with distributed reinforcement systems. In this context, high-fidelity FEA provides a more flexible and effective solution for capturing the combined effects in complex structural configurations.

FEA is an engineering analysis technique based on numerical methods. It employs discrete techniques to decompose complex structures into numerous computable small elements, solving with the aid of computers. As an efficient computational tool, FEA is widely applied in both academia and industry [30]. ANSYS Workbench is integrated software that follows FEA techniques. This software enables diverse analysis capabilities, including structural analysis, thermal analysis, and fluid analysis to calculate stress, strain, and deformation [31]. It demonstrates exceptional applicability for handling complex problems, performing coupled analyses, and optimizing designs [32]. Under the highly automated analysis platform, which offers exceptional flexibility, users can customize parameters according to special research requirements [33]. Moreover, this advanced platform provides researchers with a highly cost-effective method to create digital models and study their various performance characteristics, thereby reducing the risk of product design failures and controlling R&D costs [34]. It provides multiple finite element types for discretization and solution, including 2D solid elements (Plane 42, 182, 82/183), 3D solid elements (Solid 45, 185, 95/186, 92/187), 3D shell elements (shell 63, 93, 181, 281), and line elements (beam 3/44, 188, 189) [33]. Solid elements and shell elements are commonly used element types in ANSYS. Using them correctly enables the attainment of reasonable and accurate computational results [35].

Employing the analysis method, Singh et al. [36] investigated the natural frequencies and damping coefficients of jute-fiber- and flax-fiber-reinforced composites. Wagh et al. [37] compared the mechanical response of flax FRC with sisal FRC applied in automotive bumper beams using the FEA method. Broniewicz et al. [38] employed ANSYS software to analyze the failure characteristics of GFRP poles near the inspection hole. Xin et al. [39] used the FEA method and the experimental method to identify the buckling characteristic in composite cylindrical shells. The cylindrical shell structures have the superior capacity to bear the stresses and resist deformation, which are extensively employed in transmission pipelines, pressure vessel shells, and marine and aerospace components [40]. Huang et al. [41] reported that the form of stiffeners in a double-layer cylindrical shell structure can minimize the dynamic response at high frequency bands. Cong et al. [42] examined the buckling behavior of carbon fiber composite cylindrical shells under hydrostatic loading using experimental and numerical methods.

Hybrid composite shell hulls provide the lightweight, durable, and environmentally friendly capabilities necessary for the marine environment and represent attractive cost savings due to less fuel used, a longer range for missions, and operation costs, which are all key differentiators for defense and offshore energy [43]. Importantly, sourcing kenaf and flax locally creates new agricultural supply chains, stimulates rural economies, and generates employment opportunities [44]. Based on previous research [24,45,46], this study uses the FEA method to evaluate the effects of two hybrid composite materials applied on single-walled and double-walled cylindrical shell structures. The tested double-walled cylindrical shell structures utilize different configurations of ring and longitudinal stiffeners in combination. Their mechanical responses under various conditions are determined; the data will be assessed for the reinforcement system’s efficiency and the two hybrid composites’ performance. The von Mises equivalent stress and deformation are employed as comparative parameters to assess the structural response under different loading conditions among various structural configurations. These findings will contribute to the structural design of UMVs and advance the use of plant fiber composites in marine environments. Moreover, this study further accelerates industry adoption by providing accurate predictions of how these shells perform under bending, torsion, and hydrostatic pressure. It will also provide confidence to stakeholders and regulatory agencies with respect to performance data.

## 2. Materials and Methods

### 2.1. Raw Materials and Preparation of Hybrid Fiber Composites

This study evaluated the potential application of hybrid fiber-reinforced epoxy composites as structural materials. Epoxy resin and its corresponding curing agent were supplied by IZE Solution SDN BHD, Puchong, Selangor, Malaysia. Additionally, three plain-weave pattern fabrics as reinforcements were employed, including kenaf, flax, and glass fabrics, to enhance the composites’ physical and mechanical performance. Additionally, these fiber fabrics contain the same fiber quantity in both directions. The kenaf fabric, with a thickness of 0.68 mm and an areal density of 275 g/m^2^, was supplied by Lembaga Kenaf dan Tembakau Negara (LKTN), Kota Bharu, Malaysia. The flax fabric, measuring 0.40 mm in thickness and 223 g/m^2^ in areal density, was provided by Xuesong Weaving Factory, Jinzhou, China. The glass fiber fabric, with a thickness of 0.20 mm and an areal density of 220 g/m^2^, was obtained from Shandong Fiberglass Group Co., Ltd., Linyi, China. White nano-silica particles with a bulk density of 0.14–0.18 g/cm^3^ and a specific surface area of 170–220 m^2^/g were supplied by Beesley New Materials (Suzhou) Co., Ltd., Suzhou, China.

**Figure 1:**
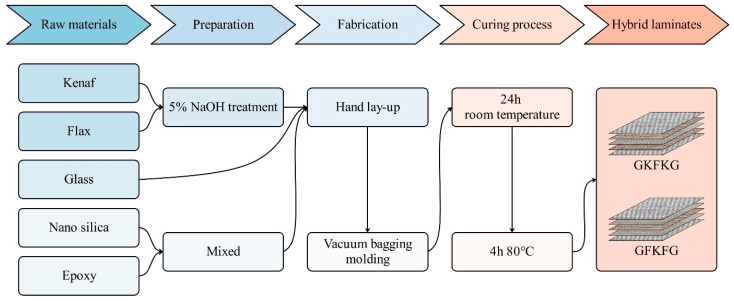
Schematic illustration of the fabrication procedure for hybrid composite laminates.

Five-layer hybrid fiber-reinforced composites were fabricated using the hand lay-up process followed by the vacuum bagging molding technique. Moreover, each layer was stacked with a [0°/90°] fiber orientation during manufacturing. A 3 wt.% nano-silica was premixed into the epoxy resin system, while the mixed matrix was then used in the fabricated laminates. Following the fabrications and curing processes (room temperature for 24 h and 80 °C for 4 h in an oven), novel hybrid laminates were successfully fabricated and possessed identical mechanical properties in both directions. The entire fabrication procedure is illustrated in Figure 1. These composite configurations were evaluated in our previous study using the analytic hierarchy process (AHP), and the stacking sequences GKFKG and GFKFG were determined to be the most promising hybrid laminates [45]. Table 1 lists the key material properties of the two hybrid composite laminates—GKFKG and GFKFG [24,45]. These data will serve as the basis for the subsequent finite element analysis.

### 2.2. Double-Walled Cylindrical Shell Configuration

**Figure 2:**
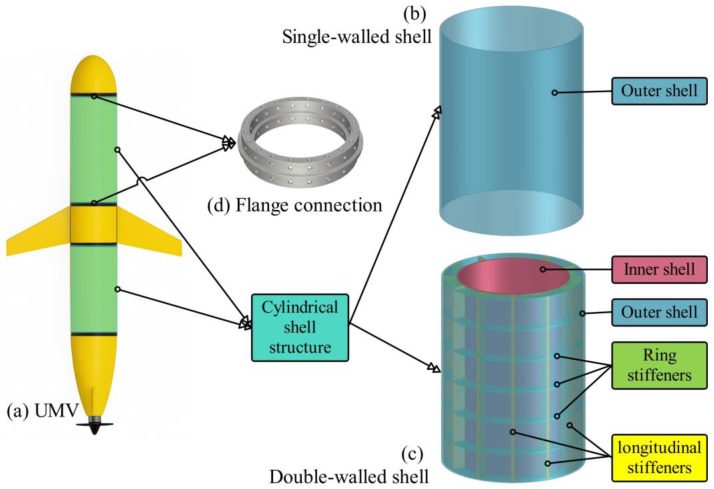
Single-walled cylindrical shell and double-walled cylindrical shell structures.

Cylindrical shell structures, due to their superior mechanical performance, low density, and high design flexibility, have been extensively employed in a wide range of engineering fields, including aerospace, civil engineering, defense systems, and sports industries [47]. Cylindrical shells are also widely employed as load-bearing structures in seawater environments, such as in submarines, autonomous underwater vehicles, torpedoes, and submarine pipelines [42]. The single-walled cylindrical shell (SS) has long been regarded as the conventional form. However, recent studies have increasingly focused attention on the structural characteristics of double-walled cylindrical shells (DSs) [48]. In this study, the focus is on a combined double-walled cylindrical shell for an unmanned marine vehicle (UMV), manufactured using hybrid composites, as illustrated in Figure 2. The shell configurations are designed to be assembled with rigid flange connections to the UMV hull. The double-walled cylindrical shell is reinforced by ring stiffeners and longitudinal stiffeners arranged between the outer and inner shells. The details of the numerical models are presented in Table 2.

### 2.3. Finite Element Model Geometry and Loading Conditions

#### 2.3.1. Finite Element Model Description

All numerical simulations are performed in ANSYS Workbench 2024 R2 software, covering the entire workflow, including geometry modeling, mesh generation, boundary and loading condition configuration, and solution procedures. The fiber orientation of composites is adjusted to be aligned along the axial or circumferential directions of the cylindrical shell, and composite materials are assumed to have orthotropic properties. To enhance computational efficiency, the SHELL181 shell element is adopted as the discretization type [38]. The element is well suited for complex thin-wall structural analysis [49]. This element is a four-node shell element, and each node possesses six degrees of freedom—three translational (UX, UY, and UZ) and three rotational components (ROTX, ROTY, and ROTZ) [50]. This study focuses on the overall mechanical response of thin-walled cylindrical shell structures under small deformations and linear elastic conditions. Using the SHELL181 element can efficiently capture the stress and deformation characteristics required for comparative evaluation among different structural configurations.

**Figure 3:**
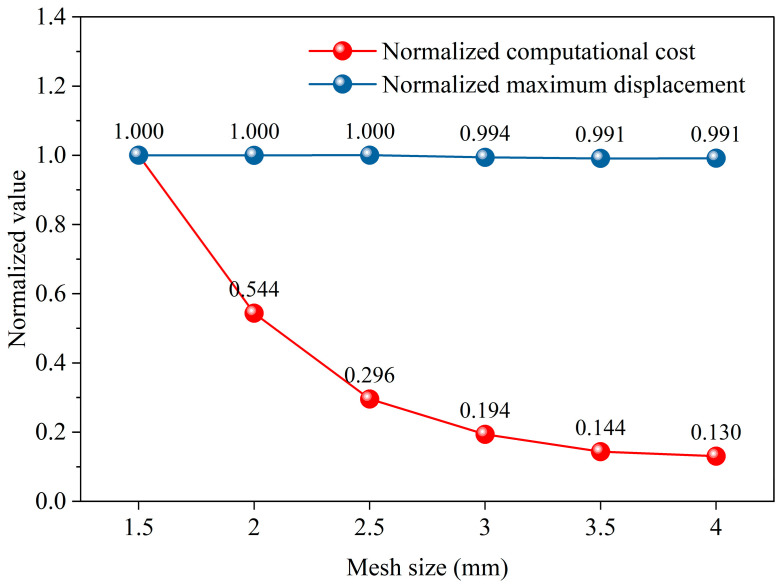
Mesh convergence study of the DSR5L12 model under hydrostatic pressure.

**Figure 4:**
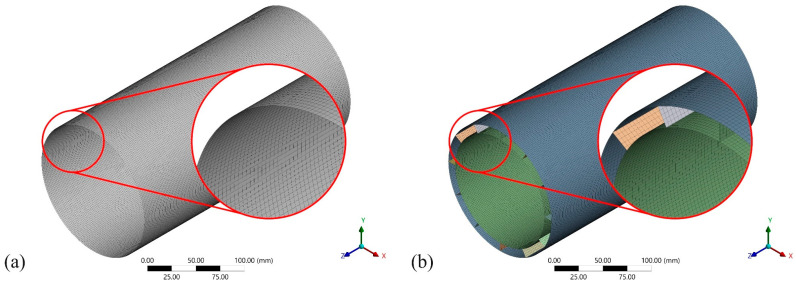
Schematic illustration of the mesh generation for the numerical models. (**a**) Single-walled cylindrical shell; (**b**) double-walled cylindrical shell (DS5R12L).

The size of the mesh directly impacts computational accuracy and efficiency [51]. To assess the numerical robustness of the finite element model, a mesh convergence study is conducted using the deformation of DSR5L12 under hydrostatic pressure as a case study. Six mesh sizes (1.5 mm, 2.0 mm, 2.5 mm, 3.0 mm, 3.5 mm, and 4.0 mm) are investigated. The maximum displacement and the consumed computational time are selected as the primary evaluation indicators. The results are normalized with respect to the finest mesh and are presented in Figure 3. With reducing mesh size, the maximum displacement gradually converges, whereas the computational time increases markedly. As a result, the mesh size of 2 mm is adopted in this study to balance the compromise between numerical accuracy and computational efficiency [52,53]. Moreover, the 2 mm quadrilateral elements are generated by implementing edge sizing and face meshing controls, which help ensure a more uniform element distribution and improved mesh quality. Finally, the total numbers of finite elements generated for SS, DS1R4L, DS2R8L, and DS5R12L are 36,000, 76,200, 80,400, and 87,000, respectively. The results indicate a positive correlation between structural complexity and the number of elements. Under the same meshing control settings (as shown in Figure 4), the double-walled cylindrical shell structures contain a larger number of calculated elements than the single-walled structure.

#### 2.3.2. Cantilever Beam with an End Point Load

**Figure 5:**
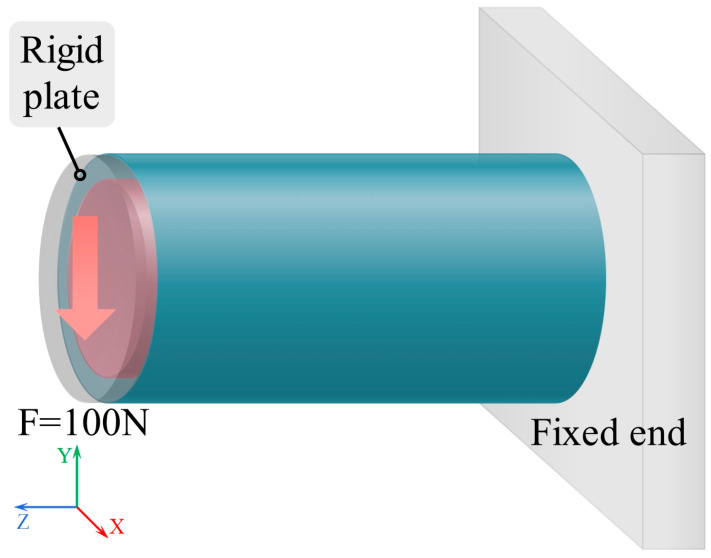
Boundary and loading conditions for the bending test.

Figure 5 illustrates the loading scheme used to evaluate the deflection and the bending stiffness of different cylindrical shells. A force of F = 100 N is applied on the free end surface of the cylinder (left end), with the direction indicated by the arrow. The opposite end (right end) is fully constrained as a fixed support, where all translational degrees of freedom (UX, UY, and UZ) and rotational degrees of freedom (ROTX, ROTY, and ROTZ) are restricted.

#### 2.3.3. Torsional Loading Condition

**Figure 6:**
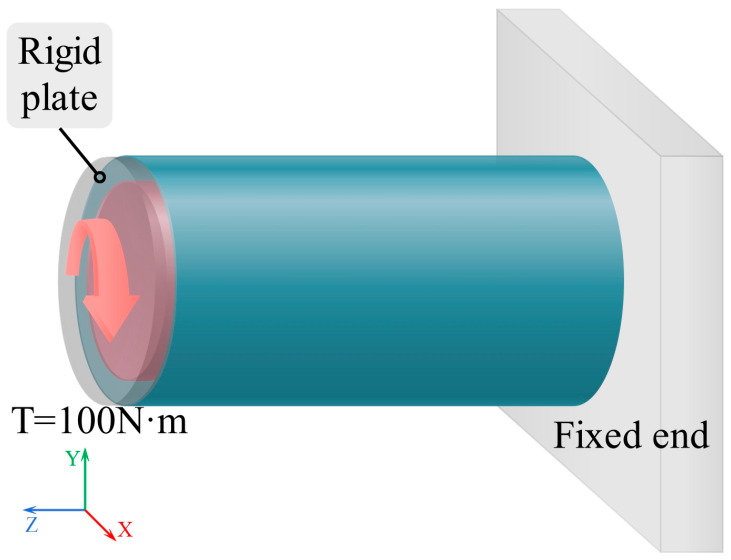
Boundary and loading conditions for the torsional test.

Figure 6 illustrates the loading configuration used to assess the torsional stiffness of the cylindrical structures. A torsional moment of 100 N·m is applied to the free end of the cylinder (left end), as indicated by the arrow. The right end is fully constrained as a fixed support, consistent with the boundary conditions adopted in the bending test.

#### 2.3.4. Structural Characteristics at a Depth of 100 Meters in Seawater

**Figure 7:**
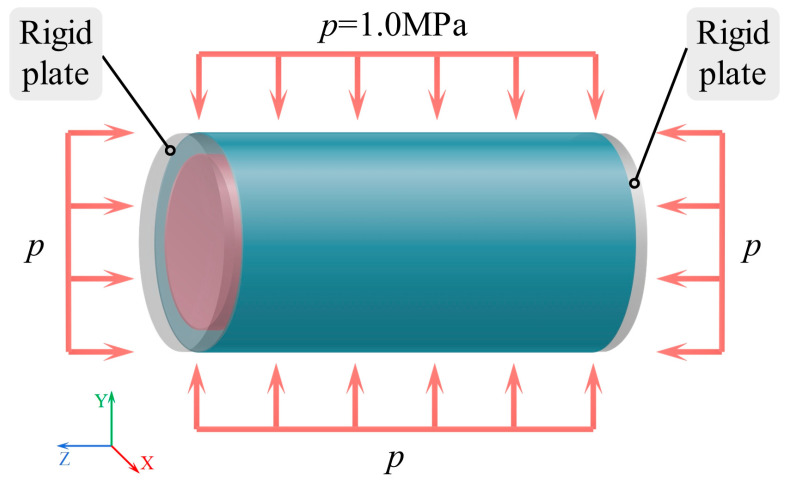
Schematic diagram of the pressure loading configuration.

Figure 7 displays the loading schematic diagram, which simulates the hydrostatic pressure acting on the cylindrical shell structure at a seawater depth of 100 m. A uniform external pressure is applied to the outer surface of the cylinder. The hydrostatic pressure is calculated using the hydrostatic relationship equation (*p* = *ρgh*), where the seawater density *ρ* = 1025 kg/m^3^, gravitational acceleration *g* = 9.8 m/s^2^, and depth *h* is 100 m. The resulting pressure is 1.005 MPa, approximately equal to 1.0 MPa, and this value will be used in the following calculation steps.

## 3. Results and Discussion

### 3.1. Bending Response

**Figure 8:**
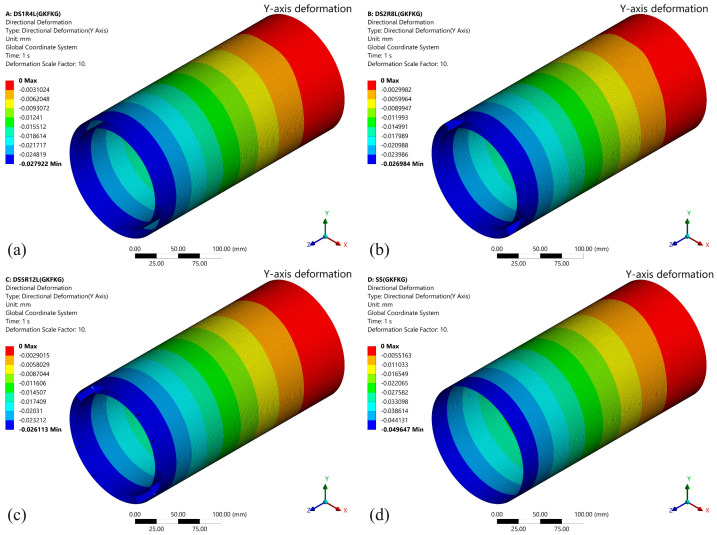
*Y*-axis deformation of the (**a**) DS1R4L, (**b**) DS2R8L, (**c**) DS5R12L, and (**d**) SS models fabricated from GKFKG composite material.

**Figure 9:**
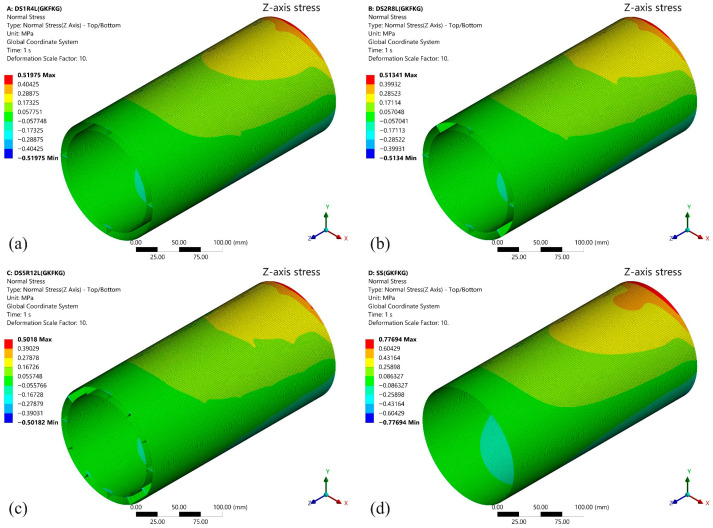
*Z*-axis stress distribution of the (**a**) DS1R4L, (**b**) DS2R8L, (**c**) DS5R12L, and (**d**) SS models fabricated from GKFKG composite material.

**Figure 10:**
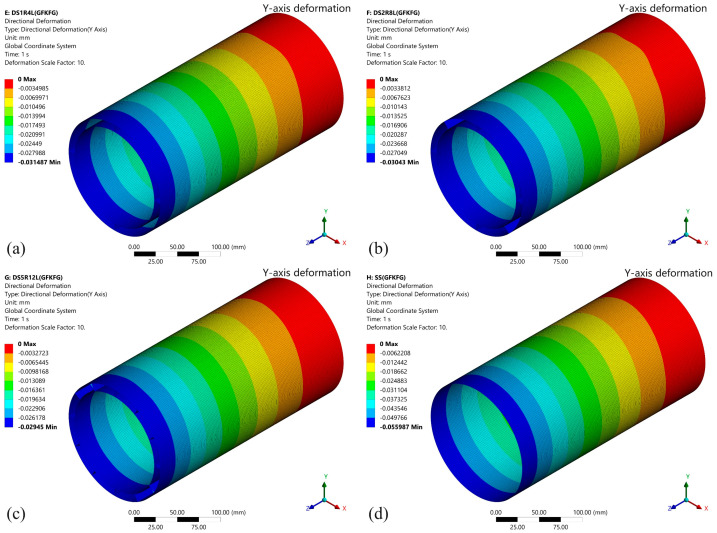
*Y*-axis deformation of the (**a**) DS1R4L, (**b**) DS2R8L, (**c**) DS5R12L, and (**d**) SS models fabricated from GFKFG composite material.

**Figure 11:**
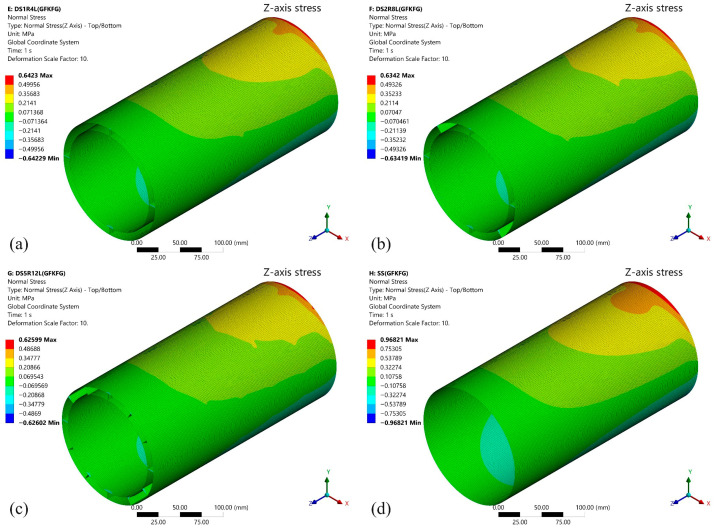
*Z*-axis stress distribution of the (**a**) DS1R4L, (**b**) DS2R8L, (**c**) DS5R12L, and (**d**) SS models fabricated from GFKFG composite material.

This section primarily examines the influence of four different configurations (SS, DS1R4L, DS2R8L, and DS5R12L) on bending response. Generally, higher bending stiffness results in smaller displacement deformation, and vice versa [54]. This characteristic plays a crucial role in overall assembly and operational stability. After calculation, the simulation results provide deformation data and *Z*-axis stress data for the four different configurations of shell structures fabricated from GKFKG and GFKFG, as shown in Figure 8, Figure 9, Figure 10 and Figure 11.

The *Y*-axis deformations under the 100 N loading force of the four models fabricated from GKFKG composite material are shown in Figure 8. It can be observed that there are significant differences in deformation resistance among different models, particularly when comparing the single-walled shell to the double-walled shell group. Under the force direction, the deformation is most pronounced in the SS, reaching 0.04965 mm, indicating its relatively weaker structural rigidity and stability. In contrast, the double-walled models (DS1R4L, DS2R8L, and DS5R12L) demonstrate superior overall deformation resistance, with the lesser deformations of 0.02792 mm, 0.02698 mm, and 0.02611 mm, indicating the double-walled structure can improve their loading behavior. When comparing the double-walled shell group, the findings reveal that altering the configurations of the ring stiffeners and longitudinal stiffeners could somewhat reduce structural deformation; however, the effect is not significant. The results imply that the effect of enhancing the double-wall structure by arranging internal cylindrical thin shells is much greater than that of ring stiffeners and longitudinal stiffeners under the bending condition. These stiffeners play a positive role in maintaining the morphological stability of the double-walled structures. Figure 9 illustrates the stress distribution along the *Z*-axis for the four structural models (SS, DS1R4L, DS2R8L, and DS5R12L) under loading. The high-stress area near the area connecting the fixed end occurs in all structures, consistent with traditional mechanical theory. The upper surface of the structure is under tensile stress, while the lower surface is under compressive stress. The SS exhibits a larger area of high-stress distribution with pronounced stress concentration. The load transfer path and stress dispersion capability in the single-wall structure are relatively limited. In contrast, the double-walled structures (DS1R4L, DS2R8L, and DS5R12L) demonstrate significant advantages in reducing stress concentration and enhancing uniform stress transfer.

Figure 10 shows the deformation along the *Y*-axis of four models made from the GFKFG hybrid composite material under a 100 N load. Similar to structures composed of the GFKFG material, the double-walled shell structures exhibit lower deformation than the single-walled shell. Under the applied load direction, the double-walled models (DS1R4L, DS2R8L, and DS5R12L) exhibited the maximum deformations of 0.03159 mm, 0.03043 mm, and 0.02945 mm, respectively. However, the SS model displays the maximum Y-direction displacement of 0.05599 mm, which is more than twice that of the double-walled models. For identical structural configurations, the maximum deflection of the GFKFG structures increased by 12.770%, 12.768%, 12.771%, and 12.779% compared to the GKFKG structures. Figure 11 depicts the *Z*-axis stress distribution of the four GFKFG structural models under loading. The maximum *Z*-axis stress values show an increase of 24.618%, 23.579%, 23.527%, and 24.744% for GFKFG composite shell structures compared to GKFKG composite structures, respectively.

The bending stiffness $K_{b}$ of the structure can be calculated using the following formulation:

(1) $$K_{b}=\frac{F}{\delta }$$

where $K_{b}$ is the bending stiffness, N/mm; *F* is the applied end force, N; and δ is the free-end deflection, mm.

**Figure 12:**
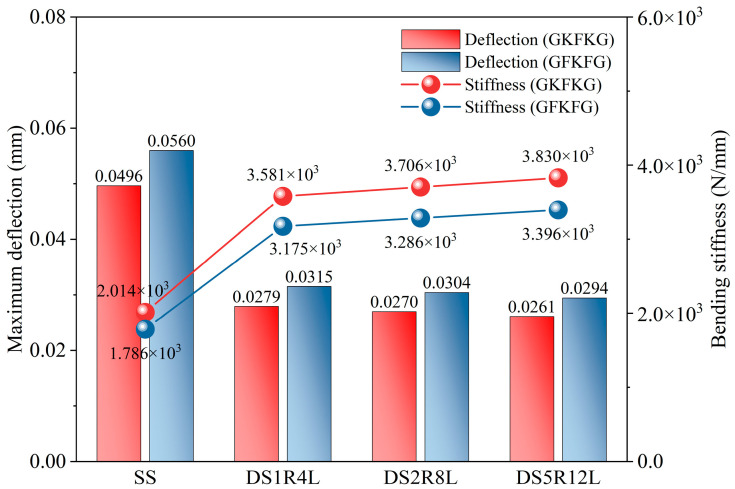
Comparison of the free-end deflections and bending stiffness of the SS, DS1R4L, DS2R8L, and DS5R12L models under an applied 100 N load.

Figure 12 compares the free-end deflection and bending stiffness of the SS, DS1R4L, DS2R8L, and DS5R12L models under a 100 N load. According to the deflection results, the SS exhibits the greatest deflection, and the deflection value progressively decreases as the structural complexity increases. This trend remains consistent across both hybrid composite materials—GKFKG and GFKFG. In addition, increasing the number of stiffeners further enhances the structure’s resistance to bending deformation. The DS5R12L model exhibits the highest bending stiffness value, reaching 3.830 × 10^3^ N/mm under the GKFKG stacking sequence composite. This is significantly higher than the SS’s value of 2.014 × 10^3^ N/mm, indicating DS5R12L requires greater load per unit deformation and possesses the strongest structural resistance to bending. The stiffness values of DS1R4L and DS2R8L fall between them. Compared with DS1R4L, the stiffness of DS2R8L and DS5R12L increases by 3.476% and 6.928%, respectively, when employing the GKFKG material; additionally, the value increases by 3.474% and 6.917% for the two forms when the GFKFG composite is used. Material selection can also affect structural bending resistance performance. Across all configurations, structures composed of GFKFG composite generally exhibit lower bending stiffness values than those made of GKFKG composite. These structures made of GFKFG composite reduce the structural bending stiffness by 11.324%, 11.322%, 11.324%, and 11.331%. The bending stiffness defined in this study represents an overall stiffness metric derived from the free-end deflection under loads at a small deflection state. This definition originates from classical cantilever beam theory and is primarily used here for comparative purposes. However, thin-walled cylindrical shell models differ from ideal beam structures. During loading, local distortion, sectional deformation, and coupling effects between multiple components in complex structures can occur. The bending stiffness may reflect the combined effects of overall bending, localized shell deformation, and sectional distortion. Therefore, the bending stiffness is intended to serve as a global comparative indicator in this study. Since all configurations adopt the same stiffness definition and are evaluated under identical loading and boundary conditions, the stiffness values still provide relative trends for assessing different structural configurations.

### 3.2. Torsional Response

**Figure 13:**
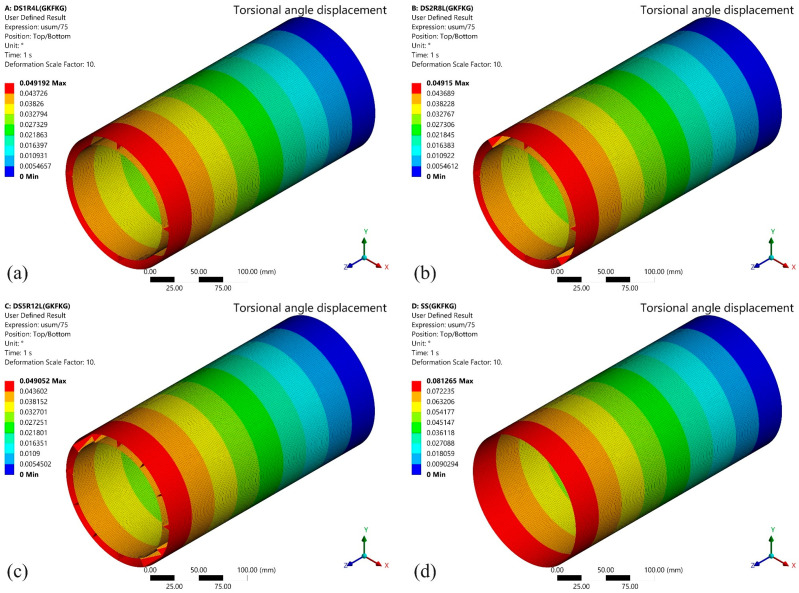
Torsional response of the (**a**) DS1R4L, (**b**) DS2R8L, (**c**) DS5R12L, and (**d**) SS models fabricated from GKFKG composite material.

**Figure 14:**
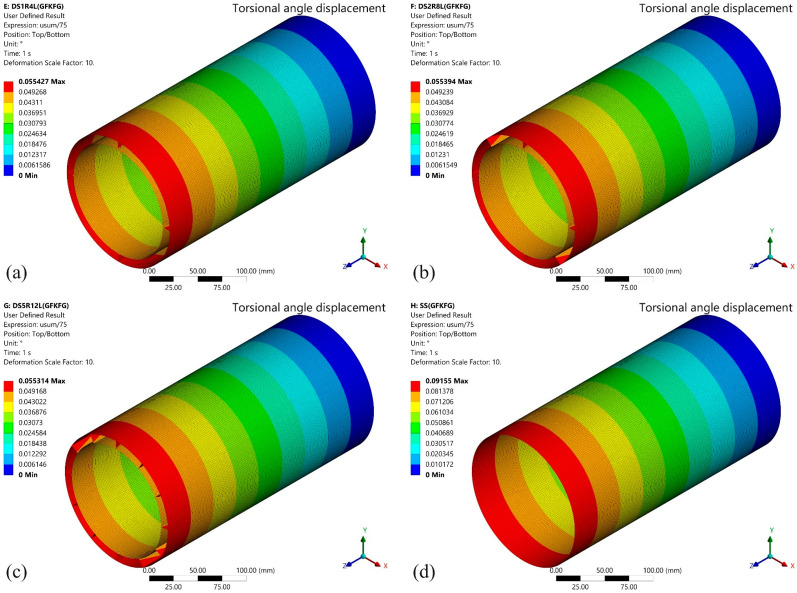
Torsional response of the (**a**) DS1R4L, (**b**) DS2R8L, (**c**) DS5R12L, and (**d**) SS models fabricated from GFKFG composite material.

Figure 13 illustrates the torsional response characteristics of the four structural models made from GKFKG composite material under torque loading. Under a 100 N·m torque, the torsional angle displacements for SS, DS1R4L, DS2R8L, and DS5R12L are 0.081265°, 0.049192°, 0.049150°, and 0.049052°, respectively. Among these, the SS exhibits the greatest torsional angle. In contrast, the double-walled group (DS1R4L, DS2R8L, and DS5R12L) exhibited significantly reduced torsional deformation. However, the double-walled structures showed nearly identical torsional angles under torque. This indicates that altering the ring and longitudinal stiffener configuration had minimal effect on resisting torsional forces. The inner and outer cylindrical walls are the primary factor in reducing torque influence. This result is also closely associated with the composition of the composite material. In the thickness of the study hybrid laminates, there are no reinforced fibers like those in the length and width directions. The longitudinal stiffeners only provide very weak stiffness in torsional moment. Additionally, the ring stiffeners cannot resist the torsional deformation; they can rotate along the centroid under the loading condition. Therefore, the stiffener system only provides weaker support to the double-wall structure in the torque. The inner and outer cylindrical walls remain the primary components that resist torsional deformation. The torsional responses of the GFKFG models under torque loading are shown in Figure 14, and the overall performance is like the GKFKG structure responses. Under torque application, the SS group exhibits a significantly larger rotation angle than the double-walled shell group, while the three structures within the double-walled group show no pronounced differences. Comparing the structural effects of the two composites reveals that the GFKFG composite exhibits slightly inferior structural performance relative to the GKFKG composite. Under the same conditions, the torsional angle of the GFKFG structure increased by 12.656%, 12.675%, 12.704%, and 12.766%, respectively, compared to GKFKG structures in the form of SS, DS1R4L, DS2R8L, and DS5R12L. The results show the improved performance of material GKFKG used in those cylindrical shell structures.

The structure’s torsional stiffness $K_{t}$ can be calculated using the following formulation:

(2) $$K_{t}=\frac{T}{\theta_{rad}}$$

where $K_{t}$ is the torsional stiffness, N·m/rad; *T* is the applied end torque, N·m; and $\theta_{rad}$ is the free-end torsional angle, rad.

**Figure 15:**
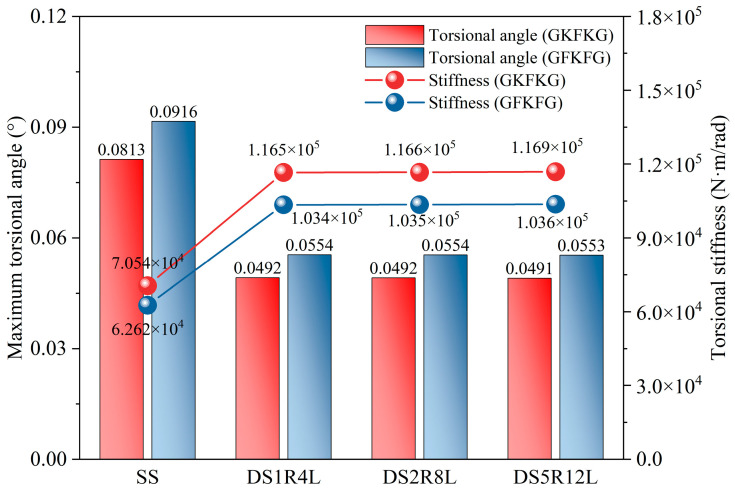
Comparison of the free-end torsional angles and torsional stiffness of the SS, DS1R4L, DS2R8L, and DS5R12L models subjected to a 100 N·m torsional load.

Figure 15 presents the comparison results (torsional angle and stiffness) of cylindrical shell structures composed of two composite materials. The double-walled structures exhibit significantly enhanced torsional stiffness, but the three different arrangements of ring and longitudinal stiffeners in the groups show limited effectiveness in contributing to the torsional stiffness. Material selection also substantially impacts structural stiffness values. Compared to cylindrical shell structures made from GKFKG composite material, the GKFKG composite structure exhibits torsional stiffness losses of 11.234%, 11.249%, 11.272%, and 11.321%. Among all comparison models, the DS5R12L double-walled shell structure made of GKFKG composite material has the highest torsional stiffness, reaching 1.169 × 10^5^ N·m/rad. However, the single-walled shell structure made of GFKFG composite material exhibits the lowest torsional stiffness value, only 6.262 × 10^4^ N·m/rad. The torsional stiffness defined in this section represents the overall torsional response under small deformation conditions. The adopted boundary conditions effectively restrain cross-sectional warping at the structural ends, thereby limiting the development of warping torsion. The reported torsional response should be interpreted as mainly applicable to cases where the shell ends are rigidly connected. Under such warping-constrained conditions, the contribution of the stiffener system to the global torsional stiffness may be reduced or weakened, and this may be the reason for the relatively small differences in torsional stiffness observed among the double-shell group.

In Section 3.1 and Section 3.2, full fixed supports are applied at the end of structures. Although the rigid boundary condition employed in bending and torsion simulations represents an idealized structural constraint, the constraint form effectively simulates the characteristics of thin-walled hull components at rigid flange connections. It should be noted that this form suppresses in-plane deformation of the edge cross-section, such as warping and ovalization. The operation may influence the absolute value of the stress and deformation response. Nevertheless, since all configurations are analyzed under identical boundary conditions, the comparative trends and relative performance remain meaningful in the scope of the research.

### 3.3. Hydrostatic Pressure Response

**Figure 16:**
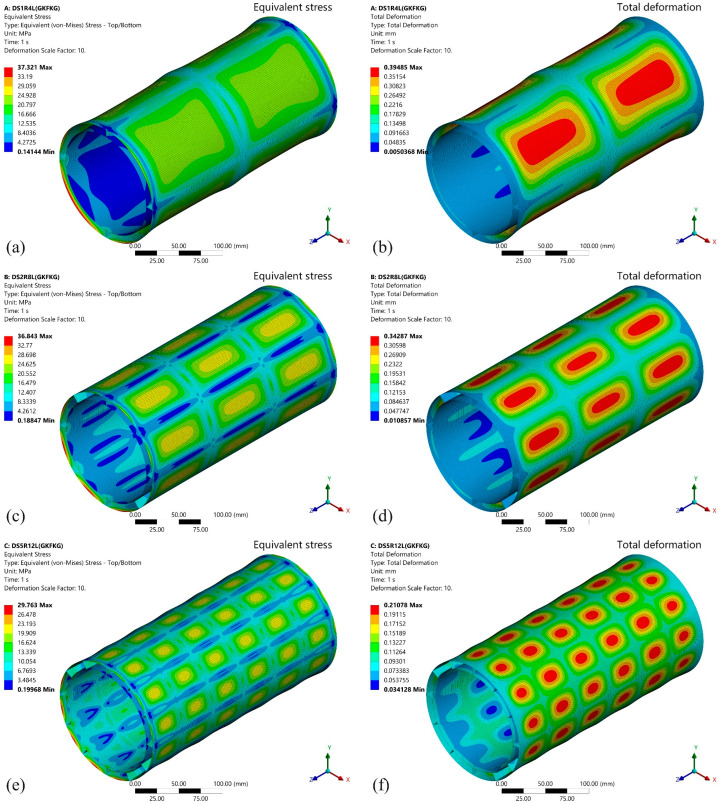
Equivalent stress and total deformation responses of the (**a**,**b**) DS1R4L, (**c**,**d**) DS2R8L, and (**e**,**f**) DS5R12L models fabricated from GKFKG composite material.

**Figure 17:**
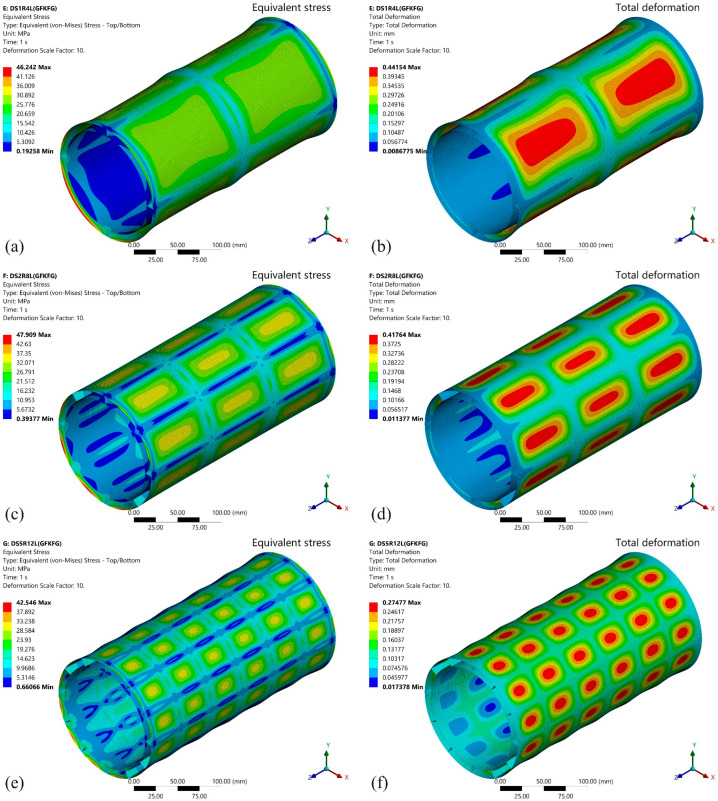
Equivalent stress and total deformation responses of the (**a**,**b**) DS1R4L, (**c**,**d**) DS2R8L, and (**e**,**f**) DS5R12L models fabricated from GFKFG composite material.

The preliminary analysis of bending and torsional performance in the preceding two sections has demonstrated the excellent capabilities of double-walled cylindrical structures. This section continues the analysis of the response of double-walled shell models (DS1R4L, DS2R8L, and DS5R12L) under hydrostatic pressure loading. Figure 16 and Figure 17 display the von Mises equivalent stress and total deformation results of the GKFKG-made and GFKFG-made structures under a hydrostatic pressure load of 1.0 MPa. The key indicators, including maximum equivalent stress and total deformation responses, are listed in Table 3.

The DS1R4L, DS2R8L, and DS5R12L exhibit significant differences in mechanical response under hydrostatic pressure. The pressure loads act on the outer wall surface, and then they are transmitted through the crossed stiffeners to the inner cylindrical wall surface. The stiffener system establishes an effective load-transfer path between the inner and outer walls, significantly enhancing structural integrity and optimizing stress distribution. This process involves the component’s collaborative load-bearing capability; the components can dissipate the pressure load and form a rational load-transfer mechanism. Increasing stiffener density subdivides the outer and inner walls into multiple small regions, effectively reducing the load-bearing area of the walls and enhancing the bending resistance of the shell structure. Moreover, the areas of high-stress zones are significantly minimized on the outer wall surface. Concurrently, the maximum total displacements decrease markedly under the three models. The results validate the rationale for optimizing the double-walled structure by adopting the fitted arrangement of ring stiffeners and longitudinal stiffeners.

In the von Mises equivalent stress plots, maximum stresses occur at the end sections on both sides. This result correlates with the rigid constraints applied at the end sections of the model. To facilitate the application of end node constraints and end pressure, rigid plates are placed at the ends of the structure. The set simulates the scenario of connecting a cylindrical shell structure with a rigid flange. Due to the combined action of the rigid flange connection and the shell structure, a locally enhanced structural configuration is formed in the ends. As a result of geometric and material stiffness discontinuities, pronounced stress concentrations occur near the region [55,56]. This observation further implies that optimal strategies should be implemented to improve connection reliability and mitigate the risk of excessive stress in thin-walled cylindrical shells. Among the GKFKG-made structures (as shown in Figure 16), the DS1R4L model exhibits a maximum equivalent stress of 37.321 MPa. The DS2R8L model shows a maximum stress of 36.843 MPa, representing a slight increase of 1.28% over the DS1R4L model. The DS5R12L model’s maximum equivalent stress decreased to 29.763 MPa, representing a substantial 20.25% reduction compared to the DS1R4L model. However, the GFKFG-made models display raised maximum equivalent stresses, as shown in Figure 17. The stresses of the DS1R4L, DS2R8L, and DS5R12L are 46.242 MPa, 47.909 MPa, and 42.546 MPa, respectively. Compared with the item of the DS1R4L model, the DS2R8L exhibits a 3.60% increase, while the DS5R12L achieves a 7.99% reduction. The results find the optimizing effect of the stiffener constructions to enhance stress distribution efficiency under the hydrostatic pressure condition. Moreover, GKFKG-made models exhibit lower maximum stresses than the GFKFG models, with reductions of 19.29%, 23.10%, and 30.05% in the item for the DS1R4L, DS2R8L, and DS5R12L models, respectively. Different stiffener configurations can impact the stress distribution. However, the finding reveals favorable performance of the DS5R12L model, which is consistent in two groups of models made of both hybrid composite materials.

Stress concentration at the structural end and stress distribution profiles under various structural configurations reveal the overall load response characteristics among different cylindrical structures under identical constraint and loading conditions. This model comprehensively evaluates the combined effects of connection style, structural configuration, and material property. Although stress concentration at ends can be influenced by the constraint and structural configurations, this indicator alone cannot indicate structural failure. Figure 16 and Figure 17 reflect the relative trends in the overall behavior of the shell structures. Both material–structure matching and structural optimization strategies should holistically consider the stress distribution across the shell while acknowledging the effects of end stresses.

Generating large deformations on the surface of the thin-walled structure is associated with insufficient material bending stiffness [57]. Moreover, according to classical mechanical principles, reducing the support span and increasing the thickness of the plates can facilitate a reduction in adverse deformation. Comparing the two hybrid laminates, GKFKG is 26.15% thicker than GFKFG. According to Formula 3 [58], the thickness is expressed in cubic form within the formula, which can significantly influence the plate stiffness more than other parameters. It is indicated that GKFKG-made models will present better resistance to pressure. The maximum deformations for all three DS models occur in the central section of the segmentation area under stiffeners. In the GKFKG-material-made group (as shown in Figure 16), the DS1R4L model, equipped with one ring stiffener positioned centrally and four uniformly distributed longitudinal stiffeners, achieves a maximum displacement of 0.3949 mm. The DS2R8L model, featuring two ring stiffeners and eight longitudinal stiffeners, has a maximum displacement of 0.3429 mm, which is 13.16% lower than the DS1R4L performance. The DS5R12L model incorporates five rings of stiffeners and 12 longitudinal stiffeners. Due to the reinforced stiffener system, it demonstrates the lowest deformation, only 0.2108 mm, a 46.62% reduction compared to the DS1R4L model. In the GFKFG-made models (as shown in Figure 17), the maximum displacement of the DS1R4L model is 0.4415 mm. The maximum displacements of the DS2R8L and DS5R12L are 0.4176 mm and 0.2748 mm, reduced by 5.41% and 37.77% compared to the DS1R4L. According to the above research results, the DS5R12L model, manufactured by GKFKG, demonstrates competitive mechanical performance under all evaluated static conditions. The combined effect of the closed stiffener system and thickened composite laminate significantly enhances the stiffness and load-carrying capacity of the double-walled cylindrical shell structure.

(3) $$D=\frac{Et^{3}}{12\left(1-\nu^{2}\right)}$$

where *D* represents the plate stiffness, N·m; *E* denotes the Young’s modulus, Pa; *t* is the thickness of the laminate, m; and ν is Poisson’s ratio.

**Figure 18:**
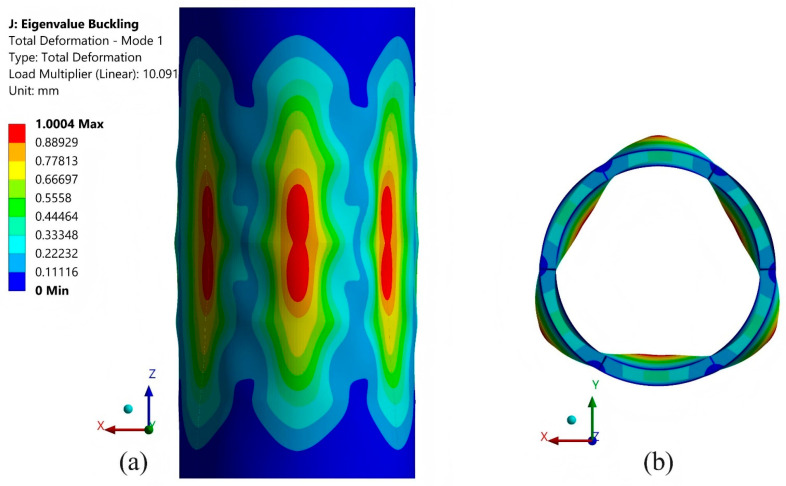
Eigenvalue buckling mode 1 of the GKFKG-made DS5R12L model: (**a**) Side view; (**b**) Top view.

**Figure 19:**
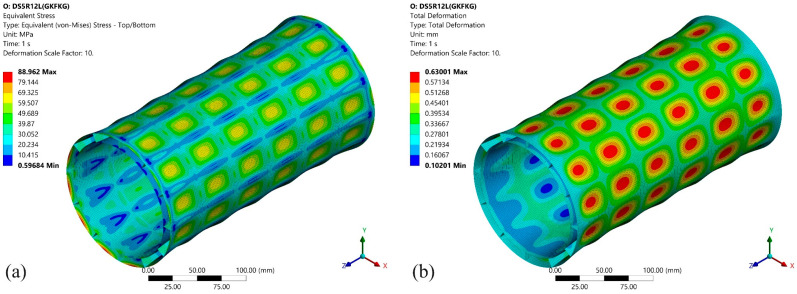
Equivalent stress and total deformation responses of the GKFKG-made DS5R12L model under 2.989 MPa: (**a**) Equivalent stress, (**b**) Total deformation.

Figure 18 presents the first eigenvalue buckling mode of the GKFKG-made DS5R2L. Pressure loadings are applied on the lateral surface of the cylindrical shell in the radial direction and the ends of the structure in the *Z*-axis direction. The critical buckling load of the model can be calculated by multiplying the applied pressure by the load multiplier. Thus, the critical buckling pressure of mode 1 is 10.091 MPa. However, eigenvalue buckling analysis for the tested shell structures may overestimate the critical buckling load due to the overly idealized nature of numerical models. This is because the models ignore initial geometric imperfections, material nonlinearity, and post-buckling behavior. Therefore, the critical pressures obtained from eigenvalue buckling analysis should be viewed as theoretical upper bounds rather than realistic failure thresholds. Moreover, under an external pressure of 2.989 MPa, the model develops the maximum equivalent stress of 88.962 MPa (as presented in Figure 19), approaching the material strength. The pressure applied at this point is significantly lower than the upper limit pressure determined by the eigenvalue buckling analysis. The hydrostatic pressure study presented here constitutes a comparative assessment conducted under idealized assumptions. Numerical simulations indicate that material strengthening and structural configuration optimization remain effective approaches for enhancing the load-bearing capacity of double-walled shell structures within the current analytical framework. Based on this context, developing advanced composite materials with superior performance can be suggested as a key research direction for further improving the load-bearing capacity of such shell structures.

This study presents a preliminary assessment of the mechanical response of three double-walled cylindrical shells fabricated from two hybrid composite materials. However, this result does not mean that the proposed models will be ready for immediate commercialization. Further experimental research and validation work are required to support practical applications, particularly in dynamic analysis, load coupling effects, and structural and material optimization. Under complex operating conditions, thin-walled cylindrical shells may suffer from in-plane sectional deformation, warping effects, and local buckling. In addition, the water absorption properties of FRCs have a significant impact on material properties, requiring special attention during use [45,59,60]. Furthermore, moisture absorption in composites also leads to morphological changes, such as swelling and degradation. For maintaining material health, advanced manufacturing processes, appropriate application of protective coatings, and standardized regular maintenance are important measures to improve the durability of composite materials [8,17]. These aspects need to be further investigated to contribute to the development and application of sustainable composite materials.

## 4. Conclusions

In engineering application areas, cylindrical shell structures are a common structural form. The structures are widely employed in marine equipment, such as submarines, torpedoes, and unmanned devices. This study investigates the bending performance, torsional performance, and hydrostatic pressure performance in a 100 m seawater environment of single-walled and double-walled cylindrical shells, which are fabricated of both kinds of kenaf-, flax-, and glass-fiber-reinforced epoxy composites (GKFKG and GFKFG). The deformation and stress distribution characteristics are systematically investigated. Through comparative analysis, the influence of material properties and structural configurations on the structures is determined, leading to the following conclusions:

1. The double-walled cylindrical shell configuration offers superior bending and torsional resistance compared to the single-walled configuration.
2. In double-wall structures, the configuration of stiffeners has a significant impact on bending stiffness but only a very slight effect on torsional stiffness. Additionally, the stiffener configuration markedly influences stress distribution and deformation under hydrostatic pressure. The DS5R12L configuration demonstrates superior performance in resisting hydrostatic pressure compared to other structural forms.
3. The type of composite material significantly impacts structural performance. Compared to structures made of GFKFG, those fabricated from GKFKG present superior performance in bending stiffness, torsional stiffness, and deep-sea conditions.
4. The DS5R2L structure fabricated from GKFKG exhibits better stability than GFKFG under pressure loading due to the double-walled form and enhanced stiffener system. The material’s intrinsic behavior will be the primary factor in its failure under high-level hydrostatic pressure.

The above findings in the research present the potential application value of hybrid fiber-based composite material (GKFKG) in the DS5R2L cylindrical shell structure. These findings also offer new opportunities for developing such structures in the UMV field, facilitating the practical expansion of kenaf/flax/glass-fiber-reinforced composite materials in the marine environment. All of this contributes to establishing hybrid biocomposite UMV hulls as a commercially viable, scalable, and sustainable solution for the global maritime marketplace.

## Figures and Tables

**Table 1:** Material properties of GKFKG and GFKFG used in the finite element analysis.

Mechanical Properties	GKFKG	GFKFG
Density (g/cm^3^)	1.27	1.28
Thickness (mm)	3.28	2.60
Fiber weight fraction (wt.%)	29.2	34.8
Tensile strength σ_1_ = σ_2_ (MPa)	88.98	103.38
Young’s modulus E_1_ (GPa)	6.04	6.81
Young’s modulus E_2_ (GPa)	6.04	6.81
Young’s modulus E_3_ (GPa)	3.11	3.11
Poisson’s ratio ν_12_	0.25	0.25
Poisson’s ratio ν_13_	0.30	0.30
Poisson’s ratio ν_23_	0.30	0.30
Shear modulus G_12_ (GPa)	2.42	2.72
Shear modulus G_13_ (GPa)	1.76	1.91
Shear modulus G_23_ (GPa)	1.76	1.91

**Table 2:** Summary of numerical models and structural configurations.

Model ID	Height (mm)	Outer Shell Diameter (mm)	Inner Shell Diameter (mm)	Ring Stiffeners (Count)	Longitudinal Stiffeners (Count)
SS	300	150	-	-	-
DS1R4L	300	150	130	1	4
DS2R8L	300	150	130	2	8
DS5R12L	300	150	130	5	12

**Table 3:** Maximum equivalent stress and total deformation responses of the DS1R4L, DS2R8L, and DS5R12L models under a hydrostatic pressure of 1 MPa.

Model ID	GKFKG		GFKFG	
	Equivalent Stress (MPa)	Total Deformation (mm)	Equivalent Stress (MPa)	Total Deformation (mm)
DS1R4L	37.321	0.3949	46.242	0.4415
DS2R8L	36.843	0.3429	47.909	0.4176
DS5R12L	29.763	0.2108	42.546	0.2748

## Data Availability

The original contributions presented in this study are included in the article. Further inquiries can be directed to the corresponding authors.
